# Clinical vignettes and global health considerations of infertility care in under-resourced patients

**DOI:** 10.1186/s40738-016-0017-6

**Published:** 2016-03-02

**Authors:** Erika Tiffanie Chow, Shruthi Mahalingaiah

**Affiliations:** 1 Department of Obstetrics and Gynecology, Boston University School of Medicine, 85 East Concord Street, Boston, MA 02118 USA; 2 Department of Epidemiology, Boston University School of Public Health, Talbot 3E, 715 Albany Street, Boston, MA 02118 USA

**Keywords:** Infertility, Under-served populations, Access to care, Patient vignettes, Refugee, Immigrant

## Abstract

The ability to receive standard of care for a diagnosis of infertility is a factor of one’s financial state and educational level, which are often correlated. Providing infertility care in an under-resourced tertiary care environment provides challenges but also opportunities for unique successes in creating a family. Among the under-represented populations are recent immigrants and refugees. Challenges arise when the infertility treatment is futile or when the standard of care is inaccessible due to cost and scheduling. Unique accomplishments are noted when families are built and hope is restored for couples fleeing from genocide and war-inflicted countries. This article will highlight two clinical vignettes from the Division of Reproductive Endocrinology and Infertility clinic at Boston University School of Medicine/Boston Medical Center. Thereafter, the article will summarize the barriers to care in the United States among those with low socioeconomic status, with non-dominant racial status (non-Caucasian), and with refugee status. All identifiers have been removed and names altered in the patient vignettes.

**Electronic supplementary material:**

The online version of this article (doi:10.1186/s40738-016-0017-6) contains supplementary material, which is available to authorized users.

## Patient Vignette 1

Mrs. AL presented to her first Reproductive Endocrinology and Infertility (REI) appointment with her husband. The couple was running late, having taken 3 different buses to arrive. When asked whether they would like to settle down, have lunch, and return at 1 PM, they replied that they did not have money for lunch. With a phone interpreter for French Creole, a full history was elicited. Mrs. AL was approximately 43 years old. She did not know her exact age but reported that the women in her family have prolonged fertility into their 60’s. She had won a lottery for a visa to the United States. She had a medical history notable for blood-loss anemia due to uterine leiomyomata. Her uterus was 24-week sized and extended laterally bilaterally. Magnetic resonance imaging was notable for multiple leiomyomata. Standard counseling regarding her fibroids, treatment of existing medical conditions, how these conditions impact fertility, age-related decline in fertility, the standard work up for infertility, and the basic self-pay costs for fertility treatments were discussed. In the intervening two years before she returned for a follow-up infertility visit, she underwent an open myomectomy where tubal patency was assessed, and she completed the basic infertility workup including ovarian function tests (diminished reserve) and semen analysis (within normal limits). Through an immersion program, she not only learned English, but she also obtained a job as a grocery bagger and saved $2000. Upon returning to clinic, she stated, “Doctor, I’m going to give you my two thousand dollars; I want that baby now.” It is apparent that despite excellent translator services and ample clinical visits, the desire to have children is very strong in some women and may cloud a true understanding of the medical complications that are involved. While multiple counseling visits (each spanning 30–45 min with an interpreter) may be beneficial in promoting health education, risk and benefit discussions, and all treatment options, the self-pay costs per visit may be a deterrent for patients to return for the clarification they need. Further, most of our online and clinical subspecialty resources are in English.

In fact, when counseling this patient on the likelihood of success and medical futility within her financial purview and comparing that with the most appropriate care for her history (which was beyond her financial means), the topic of child-free living was mentioned, and it was clear that the translator did not have the reproductive endocrinology and infertility lexicon required to translate this into French/French Creole. The translator interpreted “child-free living” as “liberté des enfants” or “liberation of the child.” The provider intended for this to be interpreted as living “sans enfants” and was able to identify the translation error despite not having fluency in French/French Creole. This patient ultimately underwent two intrauterine inseminations that were unsuccessful despite counseling on effectiveness and futility. She subsequently then presented for discussion on donor egg in vitro fertilization and potential use of a gestational carrier.

## Patient Vignette 2

LN is a 29 year-old woman who presented alone to REI clinic with a chief complaint of 2 years of infertility despite regular intercourse and monthly menstrual cycles. The interview was conducted with a phone interpreter for Amharic. The patient was very nervous and started crying when asked about family history. She was a refugee from a war-torn country where all her known family were killed in a genocide. She stated she was an only child, but it is possible that the fate of her siblings may have been too difficult to recount. Her medical history was notable for excision of a cervical lesion. She worked as a valet attendant locally, and her boyfriend worked as a valet for a local high-end hotel. She brought cash to pay for her visit and associated work-up, but she had difficulty navigating from the accounts receivable office to the lab for blood draws or to the radiology suite for her hysterosalpingogram (HSG). She was unable to complete her labs and HSG for about 3 months, and her partner was unable to complete a semen analysis due to work commitments. During her evaluation, she conceived on a clomiphene citrate challenge test and timed intercourse. Her pregnancy progressed normally until she developed preterm severe pre-eclampsia with hemolytic anemia, elevated liver enzymes, and low platelet count (HELLP) syndrome. She was delivered by cesarean section at 33 weeks gestational age with a viable infant who was discharged after a stay in the neonatal intensive care unit. Her post-partum course was complicated by being found unresponsive in her post-partum bed due to either a post-ictal state or magnesium toxicity. She was transferred to the surgical intensive care unit for monitoring and treatment of labile blood pressures. Her social support system was notable for presence of friends and community support. She and her boyfriend were married and are currently living in a safe environment. She spontaneously conceived and is expecting a second child. While this patient had social support that helped her through the complications and successful birth, many other patients presenting here do not and may even face social stigma for using infertility treatments. In addition, this patient’s social support system was able help her with her societal responsibilities, but in many cases patients who do not have the same support have to request a quick discharge from the hospital in order to return to work immediately and keep their jobs.

## Background

According to the 2002 National Survey of Family Growth, 7.4 % of married women, or about 2.1 million women, in the United States were diagnosed with infertility [1]. This number did not include surveys of women who were not married or women who were in same sex relationships who were infertile. Between 2006 and 2010, the National Survey of Family Growth reported that 14.2 % of women aged 25–44, or about 5.8 million women, had current infertility problems at the time of the survey. Of this population of women aged 25–44 with current infertility problems, 41 %, or 2.4 million women, had ever used any infertility services from 2006 and 2010 [2]. However, only 3.1 % of women aged 25–44 with current infertility problems (179,521 women) used assisted reproductive technologies (ART) in 2006–2010 [2].

Currently, 15 states offer some form of mandated infertility treatment coverage [3]. However, not everyone can afford to buy the insurance plans that would help to ameliorate the cost of infertility treatment. Even with coverage, the associated cost of ART remains high for many [2, 4, 5]. Recent literature has revealed that of the infertile women who are unable to pay for treatments, a large proportion are non-white and must deal with racial prejudices, high cost of treatment with low income, lack of understanding of medical terminology and procedures, and additional cultural expectations and stigmas [5–9]. Out of the infertile minority patient population in the United States, refugees and asylum seekers have even more difficulty accessing infertility care due to even larger language and cultural barriers, traumatic histories, and difficulty applying for cost support grants before they can be approved for permanent United States residence [10]. The United Nations High Commissioner for Refugees reports that as of December 2014, 267,222 refugees and 187,826 asylum seekers are documented in the United States [11]. It is very difficult to ascertain how many of this population are of child-bearing potential because the data is not readily available. However, of the refugee and asylum arrivals in the United States in the 2013 fiscal year, 58.6 % were aged 15–44 [12]. 46.5 % of the arrivals in 2013 were female [12]. If these percentages are extrapolated to the number of refugees and asylum seekers residing in the United States as of 2014, the potential child-bearing population is in the hundreds of thousands. It is important that these patients have access to infertility evaluation at the very least, as the evaluation may identify general health issues that converge on fertility. We outline here all the barriers to care that these populations experience in an effort to bring to light the need for changes in healthcare.

## Costs and coverage of infertility treatment

The American Society of Reproductive Medicine (ASRM) estimates that the current average cost of an in-vitro fertilization (IVF) cycle in the United States is $12,400 [13]. Even with government subsidization, the cost of an IVF cycle is around 50 % of the annual disposable income in the United States [14]. This is a huge cost to pay for the hope of having a child, yet of the 50 United States, only fifteen states mandate infertility treatment coverage. The following eleven states have a mandate to cover infertility treatments: Arkansas, Connecticut, Hawaii, Illinois, Maryland, Massachusetts, Montana, New Jersey, Ohio, Rhode Island, and West Virginia [3]. California and Texas have a mandate to offer the option to buy a health plan that covers infertility treatments; however, California does not require offering IVF coverage [3]. Louisiana and New York require that insurance companies cover the treatment of any correctable medical conditions, which includes some infertility treatments, but not IVF [3].

Under the Massachusetts State Infertility Insurance Mandate, coverage for infertility treatment is required from general insurance policies, non-profit hospital service corporations, medical service organizations, and health maintenance organizations that provide fertility-related benefits [15]. Boston Medical Center is a safety net hospital that offers underserved populations healthcare coverage under the Boston Medical Center HealthNet Plan. Safety net hospitals or hospital systems offer care without question to low-income, uninsured, and vulnerable populations [16]. HealthNet covers about 220,000 members across MassHealth (Medicaid), ConnectorCare, and Qualified Health Plans [17]. However, under the HealthNet plan, only those customers who have a Massachusetts Health Connector qualified health plan, ConnectorCare, a commercial Commonwealth Choice plan, or health insurance provided by an employer are covered for infertility treatments [18]. MassHealth, the Medicaid plan, covers infertility diagnosis and not treatment [18]. The situation is further complicated by the implementation of the Affordable Care Act, which mandates that four different plan designs be offered by health insurance companies that would cover 90, 80, 70, and 60 % of actual medical expenses depending on the price of the plan purchased [19]. Therefore, those customers that choose to purchase a health plan must decide between balancing the costs of an insurance plan and the anticipated costs of infertility treatments.

Even if a woman of child-bearing age meets the guidelines for IVF treatment in a state with mandated coverage, she will still need to make hard decisions about her choice of healthcare insurance, especially since IVF often needs repeated cycles in order to be successful. A mandate to cover infertility treatments leads to increased safer use of IVF, decreased use of riskier treatments, and higher first birth rates for women over 35 years [11, 17, 20–22]. Despite the demonstrated benefits, many states have yet to provide their citizens with mandated coverage. As a result, there is an unmet demand for infertility treatments in the United States. In 2003, only 24 % of the estimated demand for ART was met in North America [14]. The reason for this massive unmet demand, the highest amongst developed nations [14], may be due to the many barriers that infertile couples face when considering ART. Studies have shown that IVF use is closely associated with higher socioeconomic status [2, 4, 5] since the economic cost of IVF plays a large role in a couple’s decision to pursue treatment or not [23].

For refugees, the options are even more limited. Refugees can apply initially for a short-term eight month maximum health insurance called Refugee Medical Assistance, which does not cover the cost of any infertility treatments [24]. They can also apply for Medicaid or a safety net program, such as the Health Safety Net in Massachusetts [24]. However, these safety net programs only cover the cost of diagnosing infertility. If they are deemed ineligible for these programs, they will have to apply for refugee assistance from other sources or buy insurance independently through the Marketplace [24]. For this population, access to infertility care is a reach, and access to fertility preservation is an impossibility.

## Infertility treatment and race/ethnicity

In addition to financial barriers, there is overwhelming evidence that there is a huge racial/ethnic disparity amongst the demographics of patients seeking IVF treatment [5–7]. In a 561 women cohort who presented for infertility care, Caucasian women comprised a significantly larger portion of the women (80.9 %), and the African American women (4.5 %) and Hispanic women (3.9 %) who presented for care were more likely to have a lower education level and lower income [22]. A study interviewing 30 infertile Arab American men revealed that few had the means to pay for ART, even though many were offered discounts out of sympathy [10]. A survey of Latino couples, 90 % of whom immigrated from a Latin America country and 10 % of whom were born in the United States, found that none had the economic resources to pursue infertility treatment [25]. A cohort of immigrant Latinos also reported that cost, education, communication, difficulty navigating hospital and insurance systems, and poor physician care prevented them from accessing infertility treatments [8]. African American and Hispanic women were less likely than white women to have money or private insurance to help cover the costs of infertility treatments [9]. They also reported having difficulties finding a physician, taking time off of work, and paying for infertility treatments [26]. In addition, they pointed to racial/ethnic and social problems as barriers to care, such as the historic misuse of medical treatment in their communities and the stigma of infertility as perceived by friends, family, and their communities [26]. A study even found that non-white women are less likely to have insurance that would help to cover the cost of infertility treatments, further emphasizing the racial/ethnic disparity [27].

Many of the non-dominant racial/ethnic populations that struggle with infertility have lower levels of education, many face cultural barriers such as cultural stigmatization and fear of disappointing spouses and family, and many do not speak English [5, 8–10, 22, 25, 26]. To make matters worse, in 1995, the National Center for Health Statistics found that women who were non-Caucasian and had lower educational attainment reported infertility more than women who were Caucasian and had a bachelor’s degree or higher [28].

Though this is an area of developing expertise in research, the vast majority of the literature suggest poorer ART outcomes in the non-dominant racial populations – in the United States, the dominant racial population is Caucasian. In general, African American women tend to have a longer duration of infertility and higher BMI, require more aggressive ovarian stimulation, and have a higher incidence of tubal-factor infertility, uterine-factor infertility, and leiomyomata; in comparison, white women tend to have a higher incidence of polycystic ovary syndrome, endometriosis, and male-factor infertility [29]. Apart from three studies, the vast majority of the literature agrees that African American women consistently have lower success rates after ART. Amongst the outlying studies on ART outcomes in African American women, one study found more successful outcomes [30], and two studies found no significant differences between African American and Caucasian women [31, 32]. However, the Nichols et al. [30] and Bendikson et al. [31] studies dealt with cohorts with a significantly higher percentage of Caucasian women (91.9 and 91.5 % respectively), and the study conducted by Dayal et al. [32] was located in Washington DC, which the authors admitted has a large percentage of affluent African Americans, meaning that the socioeconomic factors did not modify the effect estimates as much as they would have in a more disparate neighborhood.

Fewer studies on ART outcomes in Hispanic women have been conducted, and a consensus has yet to be reached. While it has been observed that Hispanic women have a higher likelihood of having a tubal-factor infertility diagnosis and non-Hispanic white women have a higher likelihood of having an endometriosis diagnosis [33], most studies have found that there are no significant differences between the two populations in terms of birth outcomes, although Hispanic women have generally demonstrated lower utilization of infertility services [2, 34, 35].

Studies on ART outcomes in Asian women have been complicated by the fact that the group is very heterogeneous, leading some studies to group all Asian women regardless of their ethnic background and other studies to split the women into more specific racial/ethnic categories resulting in very small cohorts [29]. Asian women seem to have worse outcomes than Caucasian women with similar demographic statistics [29]. Researchers have therefore suggested that Asian race may be an independent risk factor for poor outcome after ART, although the reason for this is still unclear [36]. Genital tuberculosis, which is rare in most of the world, is an additional challenge for women who present from tuberculosis endemic areas such as India [29].

## Infertility treatment and refugees

For couples in the United States disadvantaged by low income and racial/ethnic disparities that hope to get IVF treatment, there is some hope. Organizations like the Madeline Gordon Gift of Life Foundation, the Pay It Forward Fertility Foundation, and the Tinina Q. Cade Foundation provide grants for couples that qualify as infertile according to the ASRM guidelines and are legal permanent United States residents. However, refugees and asylum seekers that have fled to the United States who struggle with infertility do not qualify for these hope grants until their application for permanent residency is approved. This population is only allowed to apply for residency once a year has passed since they entered the United States, and the duration of the application process is unpredictable.

Refugees not only have to deal with normal biological causes for infertility, but the war and hardship that they flee from also contribute to their loss of fertility due to untreated chronic illnesses that many acquire from war exposures or poor living conditions in refugee camps [10]. While refugees come from all over the globe, they universally struggle with chronic illnesses [37], mental health issues [38], untreated infectious diseases [39], nutrition-related diseases [40], substance abuse [41], and a comorbid combination of any of these conditions [42]. Refugees are further disadvantaged by having less access to necessary healthcare in their countries of origin that impact acquired causes of infertility, such as antibiotic treatments for sexually transmitted infections and corrections of fistulas that occur during and/or after unattended childbirth [43]. In addition, refugees may experience cultural stigma due to receiving infertility treatments. Only Inhorn and Fakih (2006) [10] have explored the cultural barriers that immigrants face, but we can assume that refugees and immigrants will experience similar barriers as the ones that were mentioned in the previous section in non-dominant populations. Specific cultural barriers facing Arab immigrants that Inhorn and Fakih (2006) [10] point to are: social crisis due to the inability to conceive in a pronatalist society; racial discrimination by a healthcare system that is predominantly white; and men being stereotyped as hyperfertile due to prior polygamous cultural traditions so any signs of infertility are ignored. Of the Arabs that are Muslim, they also struggle with the fact that 1) Islamic scripture disallows any form of childbearing that leads to children of unknown lineage, such as adoption or donor gametes, and 2) a belief that infertility is God’s punishment for prior sins. These barriers are likely not specific to Arab American refugee/immigrants.

While this population faces the most barriers to infertility care, they are also the least studied population when it comes to infertility care and access. A PubMed search with the terms: “infertility care refugee,” “infertility care immigrant,” “infertility access refugee,” and “infertility access immigrant” yielded only four relevant papers in total about the access to care for refugees. The three articles included in this paper described the immigrant/refugee experience in the United States. The fourth article was not discussed because it focuses on immigrants in a non-United States population (Canada), which is beyond the scope of this paper [44].

## Conclusions and future directions

Though access to infertility evaluation and treatment remains a challenge for many who face this issue, the sub-populations of immigrants and refugees face a constellation of even more adversity. Despite this, there are similarities in assumptions of what reproductive medicine can offer that transcend education and socio-economic status (Patient Vignette 1). There are unique opportunities to build families (Patient Vignette 2) and also a vast number of continued challenges.

In an effort to improve access to care for non-dominant populations, we must consider several approaches. 1) Refugees and underserved populations may be presenting for infertility care in a variety of locations from community doctor/nurse practitioner to a tertiary care center. Regardless of where they present, a full history and physical exam/medical assessment is critical in identifying any other health risks that the individual/couple may be facing. This will allow the identification of any potential health issues that converge on fertility and allow for the appropriate subsequent referrals. However, these referrals must be made with the patient’s financial and insurance status in mind. Many large hospital complexes and academic centers may not provide adequate access to the initial evaluation for these patients because either a) the patient does not have access to the appropriate insurance programs or b) the hospital does not have patient navigator services. Therefore, there needs to be a push towards outreach and collaboration with the county, community, and safety net hospitals that tend to work with this populationto ensure that the appropriate care is acquired. In addition, providers that require health insurance or a certain level of income should be prepared to offer alternatives for care. Rather than turn away a patient immediately upon discovering that the patient will be unable to pay, providers should have a list on hand of centers or clinics that the patient can try to go to for care, as well as provide them with organizations or social workers that can help the patient find a job and make enough money to save up for infertility care.

2) Another important factor is the presence of translator services in hospitals and clinics. While there is an increasing availability of translators, many of them may not be educated in the lexicon of infertility (Patient Vignette 1) and therefore need to be trained and taught the vocabulary basics of communicating not only infertility evaluation and treatment modalities, but also basic health education. Health education should not only extend to these translators however, and efforts should be made to educate non-dominant populations. These efforts are particularly important when discussing fertility treatment, especially if treatment is determined to be futile or has a poor prognosis. Patients must be counseled in all of the risks, benefits, and anticipated success of the treatment options. However, once the patients are fully aware of their situation, it is also important to respect their autonomy and treat them should they choose to receive a treatment despite futility or poor prognosis as recommended by the Ethics Committee for the ASRM [45].

3) Specific steps that can be taken to improve care for refugees include interdisciplinary care and collaborations between hospitals and refugee support systems. There is evidence that an interdisciplinary setting is more beneficial and increases access to care for refugees [46], and it is a model that needs to be further tested and implemented. At present, as part of the application for asylum, a refugee meets with a primary care physician/refugee service for a medical examination. That evaluation and point of care represents the first opportunity for identifying hardships and diagnosing chronic illnesses that a refugee could have. In cases of chronic illnesses that can cause infertility, this group must be made aware of the long term implications of leaving the illnesses untreated. Working with the communities that provide support for refugees has helped in the treatment of mental health issues [47] and should be considered as a strategy for early assessment of infertility risk.

4) Lastly and perhaps most importantly, culturally competent care towards refugees/immigrants and non-dominant populations is very necessary. One belief that converges on reproduction is that individuals or couples who are socially and economically disadvantaged do not deserve the right of access to infertility care. We must challenge this notion to better understand our individual and institutional aspects of racism. Changing this belief and expanding compassion to understand and work with extenuating circumstances that are unique to each individual patient is at the root of improving access to care. In particular, schools have begun to teach health professions students about cultural competency and the unique issues that face refugees [48]. However, more needs to be done in clinician education to include cultural competence, compassion, and understanding for the problems, wants, and needs of these deserving but underserved populations. If the status quo in healthcare can be changed and improved upon, infertility treatments will also present an opportunity to rebuild the decimated refugee populations.

In conclusion, we have utilized case presentations and literature review and propose some considerations for the approach to improving access to infertility care in vulnerable populations which have been summarized in the highlights section (Additional file 1). Despite the numerous challenges that face under-represented populations (finances, education, lack of time, cultural perceptions, comorbid health issues), it is important for providers working with this population to try their best to provide infertility care. This is particularly important not just in infertility care but also in care for these patients in all health settings. As we try to implement changes in healthcare to accommodate this population, we can hope to provide opportunities for success in creating a family.

## Ethics approval and consent to participate

Not applicable.

## Consent for publication

Not applicable.

## Availability of data and materials

Not applicable.

## Additional file

Additional file 1: Highlights of Steps to Take Going Forward. (DOCX 12 kb)

## Abbreviations

ART: Assisted Reproductive Technologies
ASRM: American Society of Reproductive Medicine
HELLP: Hemolytic anemia, elevated liver enzymes, and low platelet count syndrome
HSG: Hysterosalpingogram
IVF: In-vitro fertilization
REI: Reproductive Endocrinology and Infertility
