# Supplementary material for: Clinical vignettes and global health considerations of infertility care in under-resourced patients
Source: Fertil Res Pract. 2016 Mar 2;2:4. doi: 10.1186/s40738-016-0017-6 (PMC5424378; doi:10.1186/s40738-016-0017-6)
Supplement: Additional file 1: — Highlights of Steps to Take Going Forward. (DOCX 12 kb) [file 40738_2016_17_MOESM1_ESM.docx]

Highlights of Steps to Take Going Forward

- Full history and medical exam should be performed at first contact with each patient
- Patient education and counseling in all of the risks, benefits, and anticipated success of each treatment
- Better translator services with translators that have been trained in the lexicon
- Patient referrals must keep in mind the patient’s finance and insurance status
- Provide patients with alternative options if they do not have insurance and/or money
- Outreach and collaboration with county, community, safety net hospitals, and refugee support systems
- Training in compassion and culturally competent care
